# Quantification and Characterization of UVB-Induced Mitochondrial Fragmentation in Normal Primary Human Keratinocytes

**DOI:** 10.1038/srep35065

**Published:** 2016-10-12

**Authors:** Romain Jugé, Josselin Breugnot, Célia Da Silva, Sylvie Bordes, Brigitte Closs, Abdel Aouacheria

**Affiliations:** 1 Molecular Biology of the Cell Laboratory, Ecole Normale Supérieure de Lyon, UMR 5239 CNRS – UCBL – ENS Lyon, 46 Allée d’Italie, 69364 Lyon Cedex 07, France; 2 SILAB, ZAC de la Nau, 19240 Saint-Viance, France; 3 ISEM – Institut des Sciences de l’Evolution de Montpellier, UMR 5554, Université de Montpellier | CNRS | IRD | EPHE, Place Eugène Bataillon, 34095 Montpellier, France

## Abstract

UV irradiation is a major environmental factor causing skin dryness, aging and cancer. UVB in particular triggers cumulative DNA damage, oxidative stress and mitochondrial dysfunction. The objective of our study was to provide both qualitative and quantitative analysis of how mitochondria respond to UVB irradiation in normal human epidermal keratinocytes (NHEK) of healthy donors, with the rationale that monitoring mitochondrial shape will give an indication of cell population fitness and enable the screening of bioactive agents with UVB-protective properties. Our results show that NHEK undergo dose-dependent mitochondrial fragmentation after exposure to UVB. In order to obtain a quantitative measure of this phenomenon, we implemented a novel tool for automated quantification of mitochondrial morphology in live cells based on confocal microscopy and computational calculations of mitochondrial shape descriptors. This method was used to substantiate the effects on mitochondrial morphology of UVB irradiation and of knocking-down the mitochondrial fission-mediating GTPase Dynamin-related protein 1 (DRP1). Our data further indicate that all the major mitochondrial dynamic proteins are expressed in NHEK but that their level changes were stronger after mitochondrial uncoupler treatment than following UVB irradiation or DRP1 knock-down. Our system and procedures might be of interest for the identification of cosmetic or dermatologic UVB-protective agents.

Mitochondria are often referred to as the powerhouse of cells, generating the chemical energy (ATP) which allows eukaryotic cells to perform their essential biological functions^1^. These organelles also perform a plethora of functions besides energy production, including the regulation of cytosolic Ca^2+^ homeostasis, heme and lipid biosynthesis, intrinsic apoptosis orchestration and thermogenesis^2^. Mitochondrial dysfunction has been associated with many age-related disorders and degenerative diseases^3^,^4^, highlighting the critical importance of this organelle.

To accomplish their various tasks, mitochondria dynamically adapt their shape and distribution within the cells. Mitochondrial movement and subcellular positioning are achieved by migration along cytoskeletal tracks to reach sites of high-energy demand^5^. Mitochondrial size and shape can greatly vary according to cell type and tissue and oscillates between ‘spaghetti’-like long tubules and ‘macaroni’-like small vesicles as a result of fusion and fission events. The relative contribution of each process dictates the average size of mitochondria within the cells and their overall degree of branching. The balance of these opposing events is tightly regulated to maintain the architecture and the full metabolic capabilities of mitochondria in a wide range of conditions^6^.

In mammalian cells, mitochondrial fusion is governed by several core proteins, including mitofusin 1 (MFN1)^7^, mitofusin 2 (MFN2)^8^ and optic atrophy protein 1 (OPA1)^9^, whereas mitochondrial fission is mainly controlled by dynamin-related protein 1 (DRP1)^10^, mitochondrial fission factor (MFF)^11^ and mitochondrial fission 1 (FIS1)^12^. Homo- and hetero-dimerization between MFN1 and MFN2 are required for the tethering of adjacent mitochondria and fusion of the mitochondrial outer membrane (MOM)^8^. Although presence of MFN1 and MFN2 is needed for maintenance of normal fusion rates, evidence suggests that these mitofusins are not equivalent, MFN2 exerting pleiotropic actions in addition to its pro-fusion role^13^,^14^. OPA1 mediates fusion of the mitochondrial inner membrane (MIM) and exists in various isoforms that are produced by alternate splicing and/or proteolytic processing by mitochondrial proteases including OMA1 and YME1L^15^,^16^,^17^,^18^. OPA1-L (long) and OPA1-S (short) forms are associated with fusion and fission, respectively^18^. The dynamin-like GTPase DRP1 is thought to be predominantly localized in the cytosol from where it is translocated to the MOM to initiate mitochondrial fission. MFF may function as a DRP1 receptor upstream of FIS1 to mediate mitochondrial fission^11^. It has been shown that UV irradiation (at high dose, e.g. >100 mJ/cm^2^) and other stressors can trigger mitochondrial fragmentation (fission) in different cultured cell lines, accompanied by translocation of DRP1 and pro-apoptotic BAX to mitochondria^19^,^20^ followed by apoptosis^21^,^22^,^23^,^24^. If a mechanistic link between DRP1-dependent mitochondrial fission and BAX-dependent apoptosis is apparent, mitochondrial fragmentation has also been reported to be independent or to occur upstream of apoptosis^25^,^26^. Moreover, UV irradiation (at moderate levels) can result in mitochondrial hyperfusion rather than fragmentation^27^, a phenomenon interpreted by some investigators as a protective response^28^,^29^,^30^.

UV irradiation is a major environmental factor causing skin dryness, aging and cancer. UV irradiation (especially in the B wavelength range, 280–315 nm) triggers direct DNA damage and the production of reactive oxygen species (ROS), which also damage DNA and other intracellular constituents and induce cellular redox imbalance^31^,^32^. Changes in the mitochondrial network configuration in response to UVB irradiation has not been addressed so far in normal human epidermal keratinocytes (NHEK). The objective of the present study was to provide both qualitative and quantitative analysis of mitochondrial morphology in NHEK following UVB irradiation, with the rationale that monitoring mitochondrial alterations will give an indication of cell population fitness and enable the screening of bioactive agents with mitoprotective properties.

## Results

### UVB irradiation triggers mitochondrial fragmentation in NHEK

**Figure 1:**
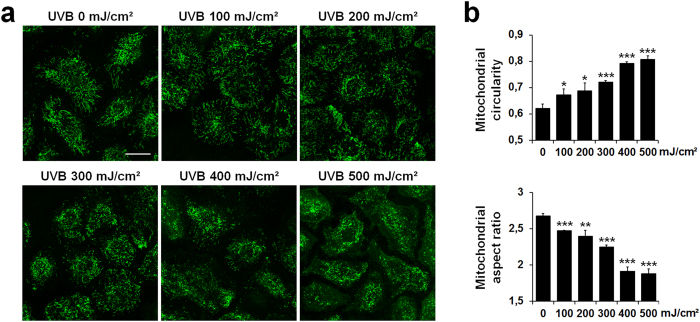
Changes in mitochondrial morphology of NHEK after irradiation with increasing doses of UVB. (**a**) Representative images of mitochondria visualized by MitoTracker Green staining in non-irradiated (control) or irradiated NHEK with indicated doses of UVB. Pictures were taken using live-cell confocal microscopy 6 h after irradiation. Scale bar = 20 μm. (**b**) Quantification of the mitochondrial shape descriptors circularity (top) and aspect ratio (bottom) using a semi-manual method. 30 cells were quantified per condition. Data represent three separate experiments on one donor and are expressed as mean ± SD. *p < 0.05, **p < 0.01, ***p < 0.001 compared with control (non-irradiated) cells using t-test.

NHEK were irradiated with increasing doses of UVB (100–500 mJ/cm^2^) and mitochondria stained with MitoTracker Green (6 h after irradiation) were visualized by live-cell confocal microscopy. UVB irradiation induced mitochondrial fragmentation as evidenced by the appearance of small round structures in cells throughout the field (Fig. 1a). Two mitochondrial shape descriptors were then measured using Fiji/ImageJ software: aspect ratio (ratio between the major and minor axes of the analyzed particles) and circularity (ratio between their area and perimeter) to estimate the length and degree of branching of mitochondria. As shown in Fig. 1b, UVB irradiation significantly increased mitochondrial circularity in a dose-dependent manner with a concomitant decrease in aspect ratio.

**Figure 2:**
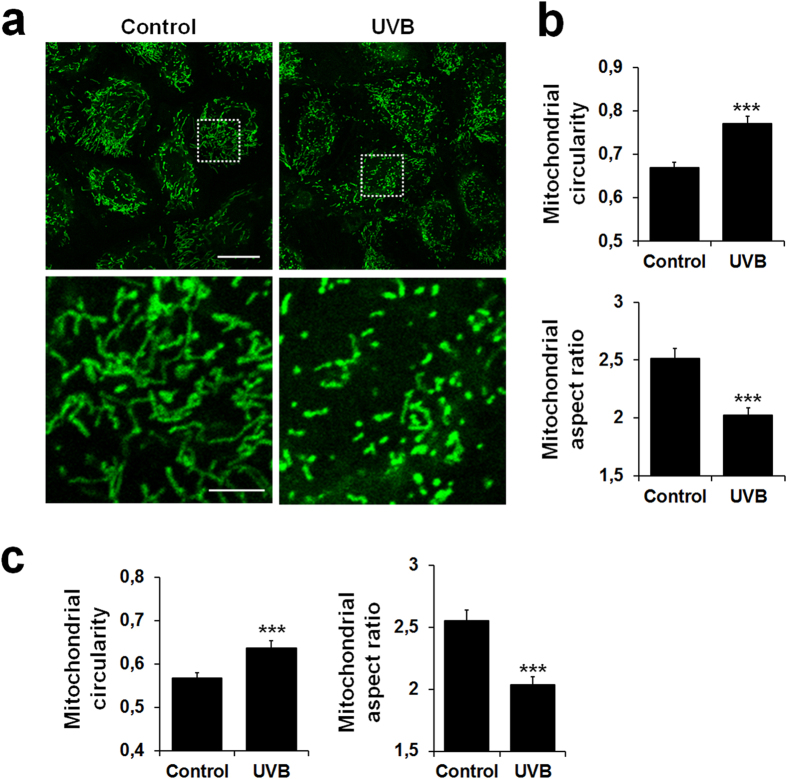
Changes in mitochondrial morphology of NHEK irradiated with a single subtoxic UVB dose. (**a**) Representative images and magnified areas of mitochondria visualized by MitoTracker Green staining in non-irradiated (control) or irradiated cells with 200 mJ/cm^2^ UVB. Pictures were taken using live-cell confocal microscopy 6 h after irradiation. Scale bar = 20 μm (magnification: scale bar = 5 μm). (**b**) Quantification of the mitochondrial shape descriptors circularity (top) and aspect ratio (bottom) using a semi-manual method. 30 cells were quantified per condition. Data represent thirteen separate experiments on five donors and are expressed as mean ± SD. ***p < 0.001 compared with control (non-irradiated) cells. (**c**) Quantification of the mitochondrial shape descriptors circularity (left) and aspect ratio (right) using our automated method (*Mitoshape*). At least 60 cells were quantified per condition. Data represent six separate experiments on five donors and are expressed as mean  ± SD. ***p < 0.001 compared with control using t-test.

Next, we sought to define a UVB dose that did not produce overt toxicity to the exposed cells while triggering mitochondrial fragmentation. This step was an important consideration in the design of assay conditions that allow screening of mitochondria-protecting agents rather than mere anti-apoptotic compounds. Based on Annexin V and propidium iodide staining (Supplementary Figure S1), a highest non-severe toxic dose was estimated at 200 mJ/cm^2^. Confocal images were recorded 6 h after irradiation, in order to visualize a clear mitochondrial fragmentation without concomitant loss of cell viability. At this dose and time interval, UVB irradiation caused a minor decrease in Δψm in comparison to treatment with the protonophore carbonyl cyanide 3-chlorophenylhydrazone (CCCP) (Supplementary Figure S2). Quantitative analysis of live NHEK from five different donors robustly established that 200 mJ/cm^2^ UVB provoked significant mitochondrial fragmentation, yielding a more punctate morphology of the mitochondria (Fig. 2a,b).

### Implementation of an automatic process for quantification of mitochondrial fragmentation

Although our semi-manual method offered accurate and reproducible measurement of changes in mitochondrial morphology, data capture was too slow and labor-intensive. Thus, we decided to implement a quantitative analysis pipeline (*Mitoshape*) to calculate the previously defined shape factors (circularity and aspect ratio) in an automated fashion. Staining with the vital fluorescent dyes CellMask Deep Red Plasma membrane Stain and Hoechst 33342 along with MitoTracker Green was used to determine cell shape and nucleus location in addition to mitochondrial morphology, which allowed computing the mitochondrial descriptors on a per-cell basis. Data were processed along the workflow outlined in Supplementary Figure S3. As shown in Fig. 2c, the automated algorithm *Mitoshape* produced similar results to those generated using the semi-manual method (Fig. 2b), allowing quick and precise assessments of UVB-induced changes in mitochondrial morphology in a quantitative and objective way. Our automatic system was also applied to measure the fragmentation of mitochondria observed after CCCP treatment (Supplementary Figure S4).

### UVB irradiation induces DRP1 mitochondrial translocation

**Figure 3:**
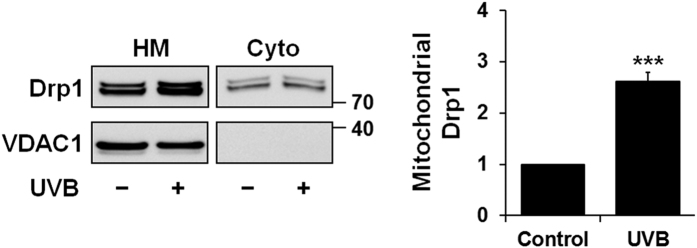
Western blot analysis of the DRP1 protein in cytosolic and mitochondrial fractions. Representative immunoblot showing subcellular distribution of DRP1 in mitochondria-enriched heavy membranes (HM) and cytoplasm (Cyto) of control NHEK (−) or NHEK irradiated (+) with 200 mJ/cm^2^ UVB for 6 h. Note that DRP1 was revealed as a doublet (but not a dimer). Mitochondrial DRP1 protein level is expressed as percentage of control. VDAC1 was used as mitochondrial marker. Data represent four separate experiments on four donors and are expressed as mean ± SD. ***p < 0.001 compared with control using t-test. The purity of the heavy membrane and cytosolic fraction was confirmed by Western blotting using an anti–VDAC1 antibody and anti-alpha-tubulin and anti-HSP70 antibodies, respectively (Supplementary Figure S8).

DRP1 exerts a crucial role in mitochondrial fission or fragmentation in a variety of models^10^,^33^,^34^. As previously described^21^,^35^, to determine whether DRP1 is involved in UVB-induced mitochondrial fragmentation in NHEK, we measured by western blot the expression level of DRP1 in mitochondria-enriched heavy membrane fractions or cytosolic fractions of NHEK cells 6 h post-irradiation with 200 mJ/cm^2^ UVB. We found that a great proportion of DRP1 adopts a mitochondrial distribution in NHEK at the steady state and that UVB irradiation increased total mitochondrial DRP1 in NHEK isolated from six of eight donors tested (Fig. 3), consistent with the observed mitochondrial fragmentation.

### Inhibition of DRP1 expression attenuates UVB-induced mitochondrial fragmentation

**Figure 4:**
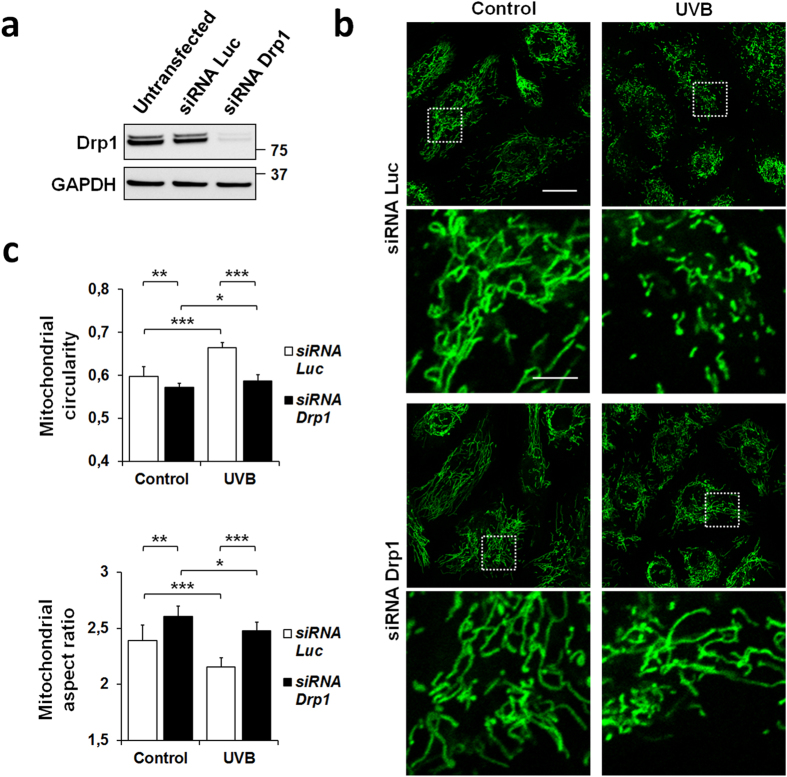
Effect of knocking down DRP1 on mitochondrial morphology in NHEK cells. (**a**) Representative immunoblot showing DRP1 protein levels in NHEK 48 h after transfection with a *luciferase* negative control siRNA or with a *DRP1*-specific siRNA. (**b**) Representative images and magnified areas of mitochondrial morphology visualized by MitoTracker Green staining in NHEK transfected with a control siRNA targeting the *luciferase* gene or with siRNA-*DRP1* and left untreated (control) or irradiated with 200 mJ/cm^2^ UVB. Pictures were taken using live-cell confocal microscopy 6 h after irradiation. Scale bar = 20 μm (magnification: scale bar = 5 μm). (**c**) Quantification of the mitochondrial shape descriptors circularity (top) and aspect ratio (bottom) using our automated method (*Mitoshape*). At least 60 cells were quantified per condition. Data represent twelve separate experiments on twelve donors and are expressed as mean ± SD. *p < 0.05, **p < 0.001, ***p < 0.001 using t-test.

To determine whether DRP1 is important for UVB-induced mitochondrial fragmentation, DRP1 expression was knocked down by transient transfection with siRNA-*DRP1*. After transfection for 48 h, high knock-down efficiency was observed for siRNA-*DRP1* by western blot analysis of total cell lysates, whereas a control siRNA against luciferase had no effect (Fig. 4a). Loss of the fission protein Dpr1 caused mitochondrial hyperfusion, which was easily detected by visual inspection of mitochondrial morphology (Fig. 4b) and confirmed using the *Mitoshape* program (Fig. 4c). Enhancement of mitochondrial fusion at baseline was induced by siRNA-*DRP1* but not the control siRNA targeting the *luciferase* gene. Moreover, UVB-induced mitochondrial fragmentation was attenuated in siRNA-*DRP1*-transfected NHEK, with a slight increase in circularity and a minor decrease in aspect ratio (Fig. 4c). In comparison, control cells preconditioned with siRNA-*luciferase* exhibited classical fragmentation rates after UVB irradiation, reflected in the measured circularity and aspect ratio shape factors. Altogether, these results indicate that expression of the endogenous DRP1 protein is important for maintaining normal mitochondrial morphology in NHEK and that loss of this factor partially alters the response of the mitochondrial network to UVB irradiation.

### UVB irradiation or DRP1 knockdown only marginally affect the levels of mitochondrial dynamic proteins

**Figure 5:**
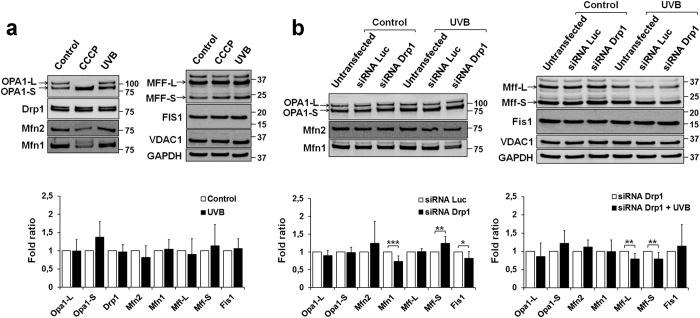
Effect of UVB irradiation or DRP1 knockdown on the levels of mitochondrial dynamic proteins. (**a**) Protein levels of mitochondrial dynamic proteins in untreated NHEK (control), NHEK treated with 10 μM CCCP or irradiated with 200 mJ/cm^2^ UVB for 6 h. Mitochondrial dynamic protein levels are expressed as percentages of control. VDAC1 was used as mitochondrial marker. Data represent five separate experiments on five donors and are expressed as mean ± SD. *p < 0.05, **p < 0.01, ***p < 0.001 compared with control cells using t-test. (**b**) Mitochondrial dynamic proteins in NHEK transfected with a control siRNA targeting the *luciferase* gene or with siRNA-*DRP1* and left untreated (control) or irradiated. Data represent eight separate experiments on eight donors. Note that MFF was found to exist in three main forms, migrating with molecular masses of approximately 25, 35 and 40 kDa. Western blot of OPA1 revealed a doublet corresponding to the OPA1-L and OPA1-S isoforms.

Next, we wondered whether UVB irradiation and/or DRP1 knockdown could affect the levels of mitochondrial dynamic proteins. The levels of the pro-fusion factors OPA1-L, MFN1, MFN2 and of the pro-fission factors OPA1-S, MFF and FIS1 were determined by western blot of total cell lysates. UVB irradiation did not alter the levels of most mitochondrial dynamic proteins (including DRP1) except for an increase in the generation of OPA1-S (Fig. 5a). DRP1 knockdown (Fig. 5b) slightly decreased expression of MFN1 and FIS1 and increased that of the short MFF isoform. UVB irradiation of siRNA-*DRP1*-transfected cells (Fig. 5b) moderately but significantly decreased expression of MFF. Interestingly, we found that UVB irradiation and CCCP treatment caused different changes in protein levels of mitochondrial dynamic actors. In CCCP-treated NHEK, the pro-fusion OPA1-L isoform completely disappeared in favor of the short pro-fission isoform OPA1-S consistent with the literature^36^, and MFN1 and MFN2 expression levels were reduced (Fig. 5a and Supplementary Figure S5).

## Discussion

Keratinocytes are repeatedly exposed to UV rays from the sun that can potentially induce damage, accelerate aging and provoke cancer^37^,^38^. UVB exposure in particular causes cumulative DNA damage and oxidative stress in irradiated keratinocytes^31^,^32^. *In vitro* culture of normal human keratinocytes (NHEK) provides a useful tool for studying how keratinocytes respond to UVB irradiation^39^,^40^,^41^,^42^,^43^. Here, we showed that NHEK undergo mitochondrial fragmentation after exposure to UVB (at doses as low as 100 mJ/cm^2^). Because mitochondrial morphology is a critical readout of cell internal state, there is a need to develop assays and tools relevant for the screening of mitoprotective agents in this cell type repeatedly exposed to high levels of UVB radiation. With this in mind, we implemented a rapid and versatile workflow (called *Mitoshape*) for the quantitative assessment of mitochondrial shape in live NHEK based on laser-scanning confocal microscopy and computational calculations of morphological parameters. Mitochondrial circularity and aspect ratio were used in the current version, but other descriptors (e.g. roundness or length) can easily be added. The *Mitoshape* tool was applied to evaluate the response of the mitochondrial network to UVB irradiation in NHEK in an unbiased and accurate manner, paving the way for future screens.

Current understanding of the regulation of mitochondrial fission indicates a key role for DRP1 in the constriction of mitochondrial membranes and organelle fragmentation^44^,^45^. Our data indicate that UVB irradiation augmented the association of DRP1 with mitochondria and that UVB-induced mitochondrial fragmentation was attenuated (but not blocked) in cells in which DRP1 expression was knocked down by siRNA transfection. The fact that NHEK mitochondria retain some potential to undergo fission in response to UVB irradiation in the absence of DRP1 suggests that DRP1 mitochondrial translocation may not be the only mechanism involved in mitochondrial fragmentation in this cell type and at the assayed UVB dose (200 mJ/cm^2^) or that fragmentation might be due to residual DRP1 expression. In case of treatment by the mitochondrial uncoupler CCCP, these variations were also accompanied by disappearance of the pro-fusion factor OPA1-L and concomitant decrease of the MFN1 mitochondrial fusion protein. The role played by DRP1 in mitochondrial fragmentation is also somewhat obscured by the mitochondrial hyperfusion phenotype observed upon knocking down DRP1 (Fig. 4b). Reduction of DRP1 protein levels in siRNA-*DRP1*-transfected cells displaced the mitochondrial fission–fusion equilibrium towards hyperfusion, generating elongated mitochondria that are probably more difficult to fragment into vesicular structures after UVB irradiation compared to control cells. Future experiments will address whether exposing NHEK to doses of UVB (e.g. 400–500 mJ/cm^2^) that lead to more pronounced mitochondrial depolarization (not shown) can enhance DRP1 mitochondrial translocation in DRP1-expressing cells and mitochondrial fragmentation in DRP1 knock-down cells.

In conclusion, we developed a novel method for automated quantification of mitochondrial morphology parameters that was used to substantiate the effect of UVB irradiation on mitochondrial shape in normal primary human keratinocytes. We also investigated the effects of UVB irradiation or DRP1 knock-down on the levels of mitochondrial dynamic proteins. We propose to employ our system and procedures for the identification of bioactive agents conferring mitochondrial protection including cosmetic or dermatologic UVB-protective agents.

## Material and Methods

### Antibodies and reagents

Primary antibodies used for Western Blotting were anti-GAPDH (MAB374; Millipore), VDAC1 (ab14734; Abcam), MFN1 (ab57602; Abcam), MFN2 (ab56889; Abcam), OPA1 (612607; BD Biosciences), FIS1 (10956-1-AP; Proteintech), MFF (17090-1-AP; Proteintech), DRP1 (Dlp1; 611112; BD Biosciences), involucrin (I9018; Sigma), profilaggrin (sc66192; Santa Cruz), HSP70 (ADI-SPA-810; Enzo life science), alpha-tubulin (sc8035; Santa Cruz). The following secondary antibodies were used: horseradish peroxidase-conjugated rabbit anti-mouse (P0161; DAKO) and horseradish peroxidase-conjugated goat anti-rabbit (PO448; DAKO). Hepes, D-Mannitol, Sucrose, Ethylenediaminetetraacetic acid (EDTA), Trizma Hydrochloride (Tris-HCL), Sodium chloride (NaCl), Igepal (NP-40), Sodium deoxycholate (DOC), Sodium dodecyl sulfate (SDS), Carbonyl cyanide m-chlorophenyl hydrazone (CCCP) were purchased from Sigma Aldrich.

### Cell culture and treatment

NHEK were obtained from SILAB S.A (Saint-Viance, France) from redundant skin of healthy donors undergoing abdominoplasty or reduction mammoplasty. NHEK were cultured at 37 °C in a humidified atmosphere containing 5% CO_2_ in Keratinocyte Serum Free Medium (KSFM, Life technologies) supplemented with 25 μg/mL Bovine Pituitary Extract (BPE, Life technologies), 1.5 ng/mL Epidermal Growth Factor (EGF, Sigma Aldrich), 500 U/mL penicillin/streptomycin (Life technologies) and 0.25 μg/mL fungizone (Life technologies). Cells were plated at 3 × 10^5^ cells per well 48 h before treatment with CCCP at 10 μM for the indicated time or UVB irradiation at the indicated dose and time. Western blot analysis of terminal differentiation markers (i.e., involucrin, profilaggrin) indicated that these cells were undifferentiated human keratinocytes (Supplementary Figure S6). Cells were seeded to reach approximately 80% confluency at the time of treatment or irradiation. UVB irradiation was performed using a BS-02 UV irradiation chamber and UV-Mat dosimeter (Dr. Gröbel UV-Elektronik GmbH, Ettlingen, Germany). The lamp emits UVB irradiation with a peak at 311–312 nm (see Supplementary Figure S7 for spectrum) and partially excludes shorter wavelengths, such as UVA.

### Western Blot

Whole-cell extracts were prepared in RIPA Buffer (50 mM Tris pH 7.5, 150 mM NaCl, NP40 1%, sodium deoxycholate 0.5%, SDS 0.1%) supplemented with protease inhibitor cocktail (Roche). Cells were lysed and incubated on ice for 20 min and then centrifuged at 15,000 g for 30 min to remove cell debris. Supernatants were collected and protein assays were performed using BCA assay kit (Sigma Aldrich). Equal amount of proteins were resuspended in 4X LDS Buffer (Life technologies) and 10X sample reducing agent (Life technologies), boiled for 10 min at 95 °C and resolved by SDS-PAGE with precast NuPAGE Novex 4–12% Bis/Tris gradient gels in MES Buffer. Separated proteins were transferred to a nitrocellulose membrane using iBlot dry blotting system (Life technologies) according to manufacturer’s protocol. After incubation with 5% non-fat milk in PBS containing 0.05% Tween 20 (Euromedex), membranes were probed with primary antibodies overnight at 4 °C. Immunoreactive complexes were detected after 1 h incubation with secondary antibodies at room temperature using Luminata Forte Western HRP substrate (Millipore) or Supersignal West femto Chemiluminescent substrate (Fisher scientific) depending on immunoblot sensitivity.

### siRNA transfection

For human *DRP1* RNAi, 27-base nucleotides were chemically synthesized (Eurogentec): *Dnm1L-F*: 5′-ACUAUUGAAGGAACUGCAAAAUAUA-3′ and *Dnm1L-R*: 5′-UAUAUUUUGCAGUUCCUUCAAUAGU-3′. *Luciferase* RNAi was used as control: *Luc. GL3-F*: 5′-CUUACGCUGAGUACUUCGA-3′ and *Luc. GL3-R*: 5′-UCGAAGUACUCAGCGUAAG-3′. NHEK were plated in 6-well plates or in 35 mm petri dish (at 3.5 × 10^5^ cells per well) and simultaneously transfected with 1 nM of the indicated siRNA using 4 μL Lipofectamine RNAiMAX (Life technologies). Cells were treated or irradiated 48 h post-siRNA transfection.

### Quantitative assessment of mitochondrial membrane potential by fluorescence-activated cell sorting

Δψm was quantitatively monitored using TMRM dye (Life technologies) as previously described^46^. NHEK were plated in 12-well plates (at 1.2 × 10^5^ cells per well) 48 h prior to staining with TMRM. Cells were incubated with 600 nM TMRM diluted in culture medium for 30 min at 37 °C in a humidified atmosphere containing 5% CO_2_. Cells were then rinsed in DPBS and incubated in culture medium containing 150 nM TMRM. All treatments were performed in the presence of 150 nM TMRM. Following treatments, cells were trypsinized, rinsed in DPBS and then kept on ice. Flow cytometric measurements were performed with a BD LSRII flow cytometer (BD Biosciences). Data were analyzed using FlowJo software.

### Annexin V/Propidium Iodide assay

Apoptosis assay was performed by flow cytometric analysis of cells double stained with Annexin-V-FITC and Propidium iodide (PI). Cells were plated in 6-well plates (at 3 × 10^5^ cells per well) 48 h prior treatment. Treated NHEK were stained using Annexin-V-FLUOS staining kit (Roche) according to the manufacturer’s instructions. Flow cytometric measurements were performed as described above.

### Cytosolic and mitochondrial fractionation

To extract cytosolic and mitochondrial fractions, cells were homogenized and mitochondria pelleted as previously described^47^. Briefly, treated cells were collected, washed in ice-cold PBS and the following isolation procedure was carried out at 4 °C. Cells were suspended in Mitochondrial Buffer (MB) (10 mM Hepes pH 7.5, mannitol 210 mM, sucrose 70 mM and EDTA 1 mM) supplemented with protease inhibitor cocktail (Roche) and lysed using two rounds of 60 strokes of a 2 mL glass/glass dounce homogenizer (Kontes). The homogenates were centrifuged at 1,500 g for 5 min to discard nuclear pellet and debris. The remaining supernatant was centrifuged at 10,500 g for 5 min to obtain a cytosolic fraction in the supernatant. The pellet containing the heavy mitochondrial fraction was lysed in RIPA Buffer (50 mM Tris pH 7.5, 150 mM NaCl, NP40 1%, DOC 0.5%, SDS 0.1%) with protease inhibitor cocktail (Roche) and incubated on ice for 20 min. The extracted mitochondrial proteins were stored at −80 °C for further analysis.

### Cell staining for confocal microscopy

Cells were plated in 35 mm petri dish with cover glass bottom thickness 170 μM (CG 1.5) at 3 × 10^5^ cells per dish and cultured in medium until they approximately reached 80% confluency at time of treatment or irradiation. NHEK after treatment were stained in culture medium containing 500 nM MitoTracker Green FM (Life technologies) and 2 μg/mL Hoechst 33342 (Sigma Aldrich) for 30 min at 37 °C. After being loaded, cells were washed twice with PBS and replaced at 37 °C before live-cell imaging. Immediately before imaging, cells were pulsed with 5 μg/mL CellMask deep red plasma membrane stain (Life technologies) for 5 min at 37 °C.

### Confocal fluorescence microscopy and image analysis

Images were acquired on a Zeiss LSM710 confocal inverted microscope with a 63 × 1.42 NA objective. Hardware and image acquisition were controlled by Zen 2012 software. Quantitative analysis of mitochondrial morphology was performed on confocal images using two different protocols. A semi-manual method was based on the manual delimitation of the cells using Fiji/ImageJ software. An automatic method used Matlab software to create a mask for each cell after Hoechst 33342 (for nuclei) and CellMask deep red plasma membrane stain (for cytoplasm) stainings. In both cases, confocal image of MitoTracker Green fluorescence were processed, binarized and subjected to particle analysis to generate mitochondrial morphological characteristics for each cell: length or aspect ratio (AR, ratio between the major and minor axis of the ellipse equivalent to the mitochondrion) and degree of branching or circularity.

## Additional Information

**How to cite this article**: Jugé, R. *et al.* Quantification and Characterization of UVB-Induced Mitochondrial Fragmentation in Normal Primary Human Keratinocytes. *Sci. Rep.* **6**, 35065; doi: 10.1038/srep35065 (2016).

## Supplementary Material

Supplementary Information
