# Supplementary material for: Quantification and Characterization of UVB-Induced Mitochondrial Fragmentation in Normal Primary Human Keratinocytes
Source: Sci Rep. 2016 Oct 12;6:35065. doi: 10.1038/srep35065 (PMC5059735; doi:10.1038/srep35065)
Supplement: Supplementary Information [file srep35065-s1.pdf]

QUANTIFICATION AND CHARACTERIZATION OF UVB-INDUCED MITOCHONDRIAL FRAGMENTATION IN NORMAL PRIMARY HUMAN KERATINOCYTES

Romain Jugé [1,2], Josselin Breugnot [2], Célia Da Silva [1], Sylvie Bordes [2], Brigitte Closs [2] and Abdel Aouacheria [1,3]*

[1] Molecular Biology of the Cell Laboratory, Ecole Normale Supérieure de Lyon, UMR 5239 CNRS – UCBL – ENS Lyon, 46 Allée d'Italie, 69364 Lyon Cedex 07, France

[2] SILAB, ZAC de la Nau, 19240 Saint-Viance, France

[3] ISEM - Institut des Sciences de l'Evolution de Montpellier, UMR 5554, Université de Montpellier | CNRS | IRD | EPHE, Place Eugène Bataillon, 34095 Montpellier, France

*Corresponding author: Abdel Aouacheria, ISEM CNRS UMR 5554, Université de Montpellier, Place Eugène Bataillon, 34095 Montpellier ; France Phone: +334 67 14 46 88; Fax: +33 4 67 14 36 22 ; abdelouahab.aouacheria@umontpellier.fr; aouacheria.abdel@gmail.com

Short title: Mitochondria in UVB-irradiated keratinocytes

Abbreviations: CCCP, Carbonyl cyanide 3-chlorophenylhydrazone; Drp1, Dynamin-related protein 1; NHEK, Normal human epidermal keratinocytes

Supplementary Information

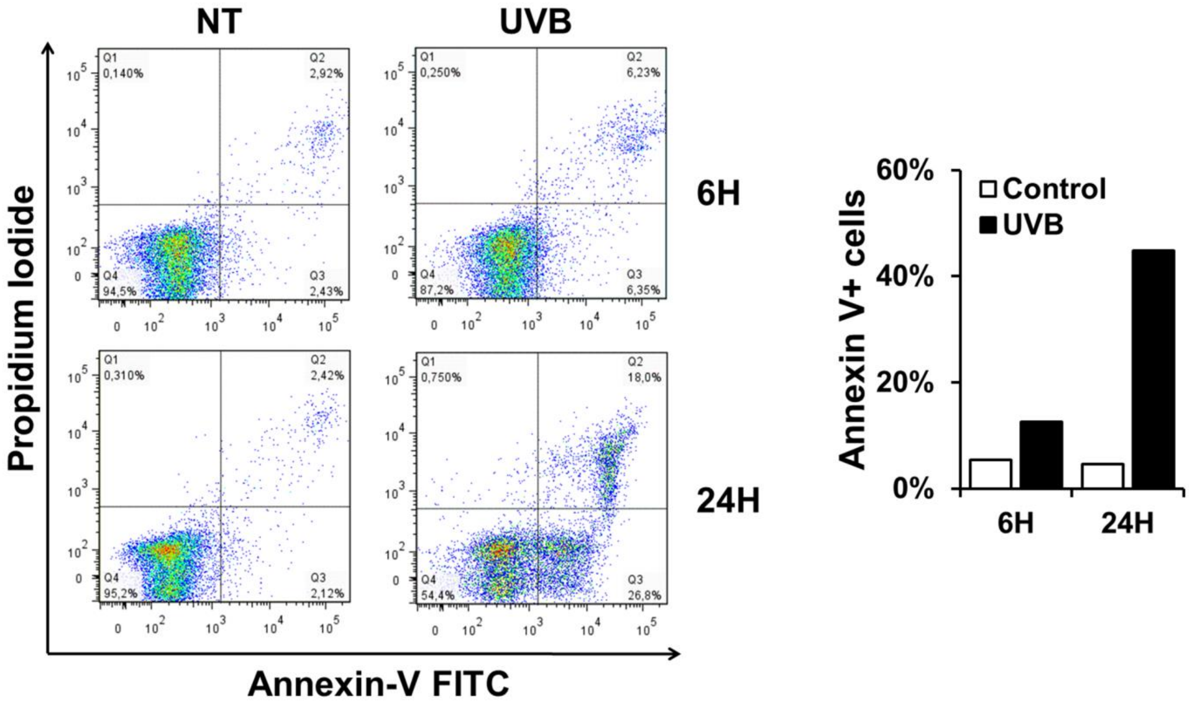

Supplementary Figure S1. Assessment of cell death by annexin V and PI staining. Apoptotic cells were assessed by flow cytometry using Annexin V and propidium iodide (PI) staining on non-irradiated NHEK (control) or NHEK irradiated with 200mJ/cm² UVB, 6h or 24h post-irradiation. Left: representative flow cytometry graphs. Right: percentage of apoptotic cells stained with annexin V. The experiment was performed in triplicate.

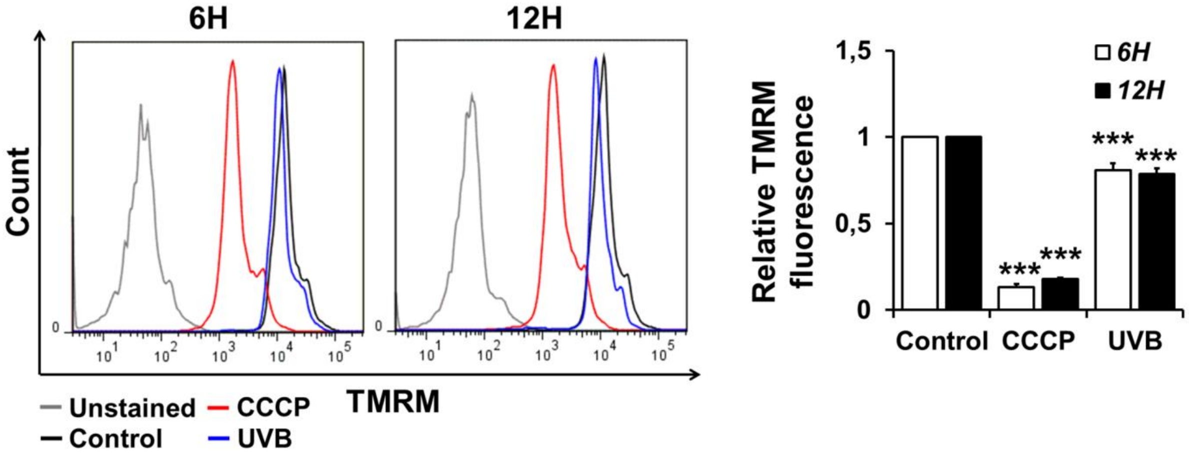

Supplementary Figure S2. Mitochondrial membrane potential analysis by FACS. Representative flow cytometry graphs showing the intensity of TMRM signals in untreated NHEK (control), in NHEK treated with 10µM CCCP or irradiated with 200mJ/cm² UVB for 6 or 12h. The bar graph shows quantification of TMRM signals measured by FACS analysis and expressed in percentage compared with control. Data represent three separate experiments on three donors and are expressed as mean ± SD. *** p<0.001 compared with control using t-test.

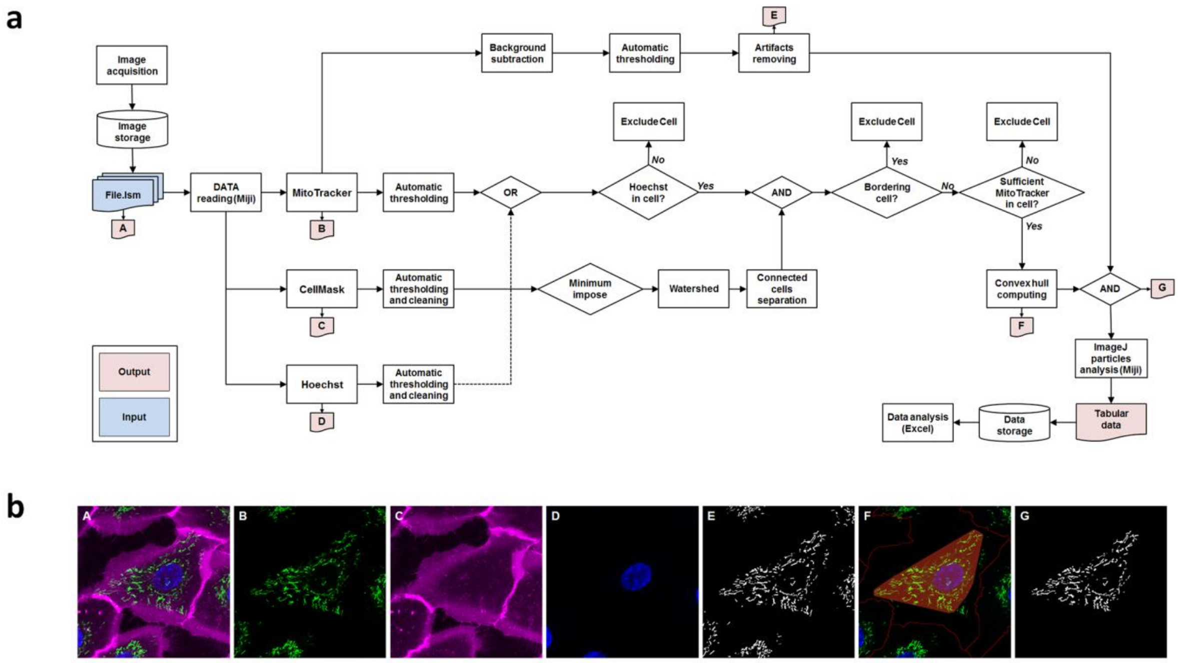

Supplementary Figure S3. Mitoshape workflow. (a) Mitochondrial morphology was analyzed by combining Matlab image processing algorithms and Fiji/ImageJ tools using the Miji package. Hoechst channel was used to determine which pixels belong to a nucleus and which ones do not. As each cell is assumed to have a nucleus, Hoechst staining permits to quantify mitochondrial parameters only in regular cells (while discarding artefacts). A disconnection of neighboring or contacting cells is performed using the Cellmask channel and a watershed algorithm. Area borders are expanded so that they will correspond with the physical limits of the cell. Truncated cells that touch the borders of the imaging area are rejected, as well as those presenting too few staining. Location of Region of Interest (ROI) was used for quantitative evaluation of mitochondrial circularity and aspect ratio per cell using Fiji/ImageJ algorithm. Automatic thresholding was selected using Otsu's method[1]. (b) Representative images of each step of the *Mitoshape* process labelled as an output in (a).

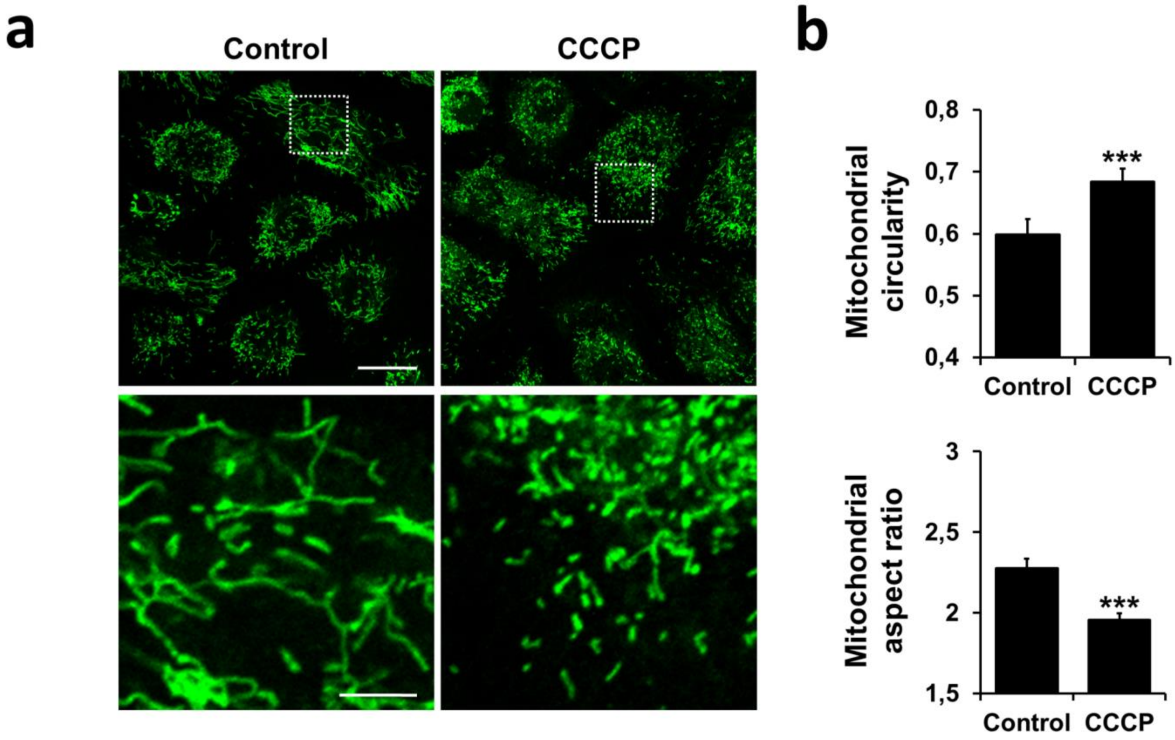

Supplementary Figure S4. Changes in mitochondrial morphology of NHEK treated with the protonophore CCCP. (a) Representative images of mitochondria visualized by MitoTracker Green staining in non-treated cells (control) or cells treated with 10µM CCCP. Pictures were taken using live-cell confocal microscopy 6h after irradiation. Scale bar = 20µm. (b) Quantification of the mitochondrial shape descriptors circularity (top) and aspect ratio (bottom) using our automated method (*Mitoshape*). At least 60 cells were quantified per condition. Data represent eight separate experiments on six donors and are expressed as mean ± SD. *** $p<0.001$ compared with control using t-test.

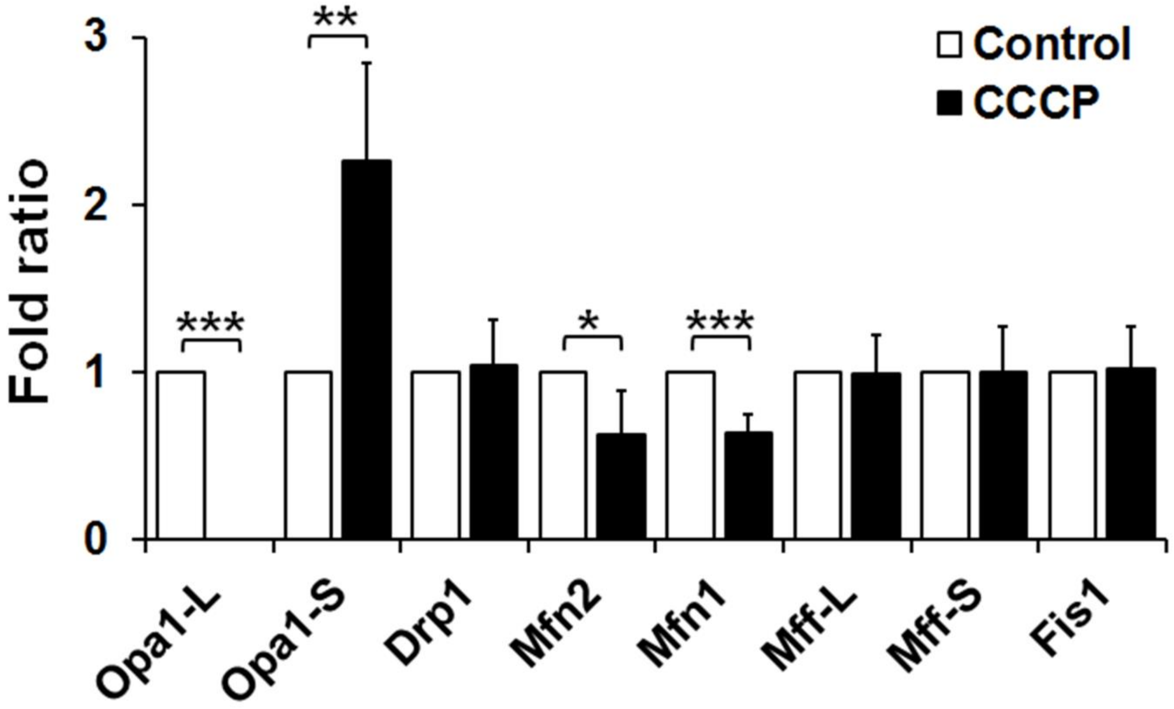

Supplementary Figure S5. Effect of CCCP treatment on the expression of mitochondrial dynamic proteins. Mitochondrial dynamic protein levels in untreated NHEK (control) or NHEK treated with 10µM CCCP expressed as percentages of control. VDAC1 was used as mitochondrial marker. Data represent five separate experiments on five donors and are expressed as mean ± SD. * $p<0.05$, ** $p<0.01$, *** $p<0.001$ compared with non-treated cells using t-test.

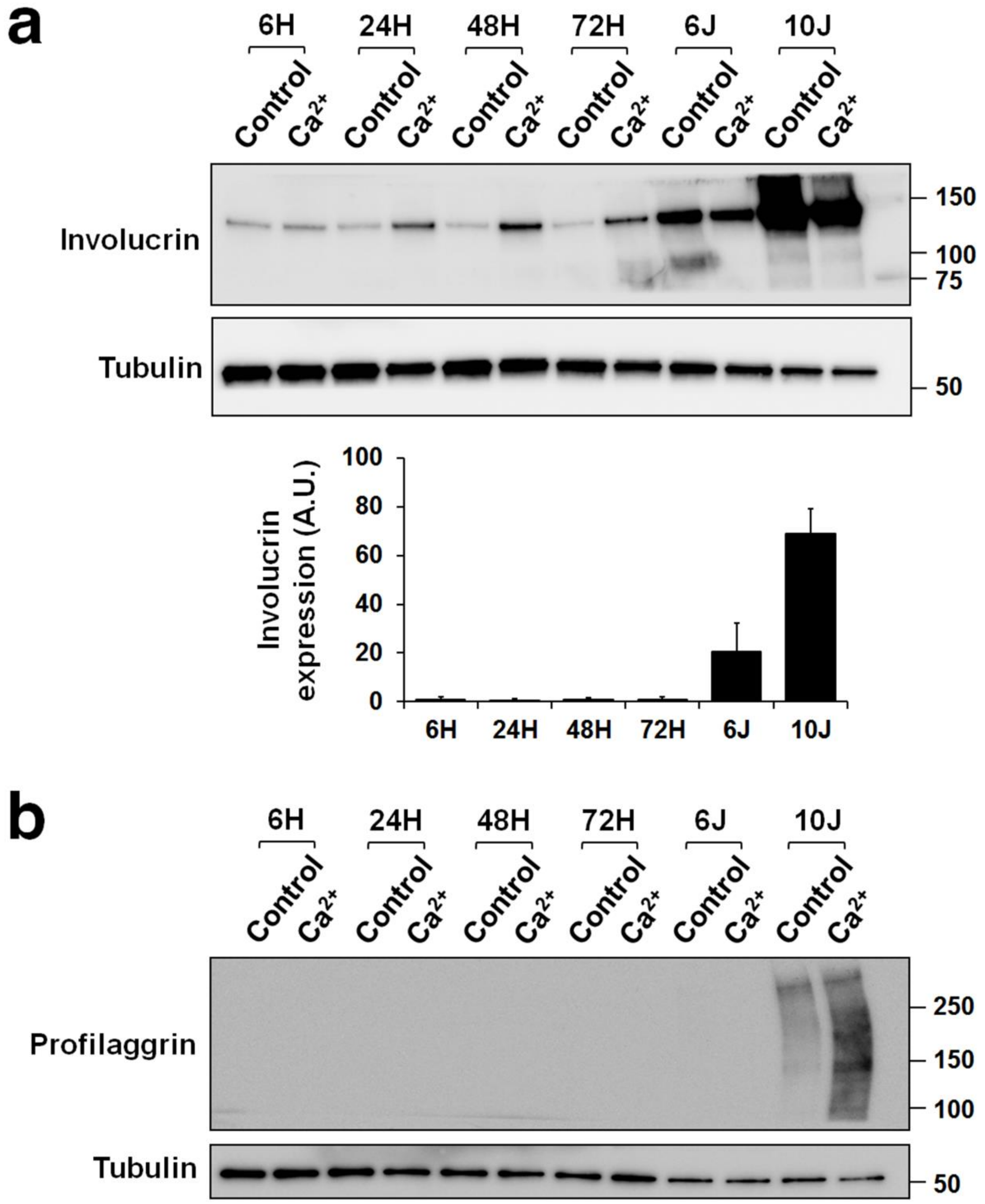

Supplementary Figure S6. Western blot analysis of terminal differentiation markers in cultured epidermal cells. The cells used in the work (P3, 48h after initiation of the culture) express negligible amounts of involucrin (a) and profilaggrin (b), which are early and late stage keratinocyte differentiation markers, respectively. Data represent three (a) or two (b) separate experiments on two different donors of various ages.

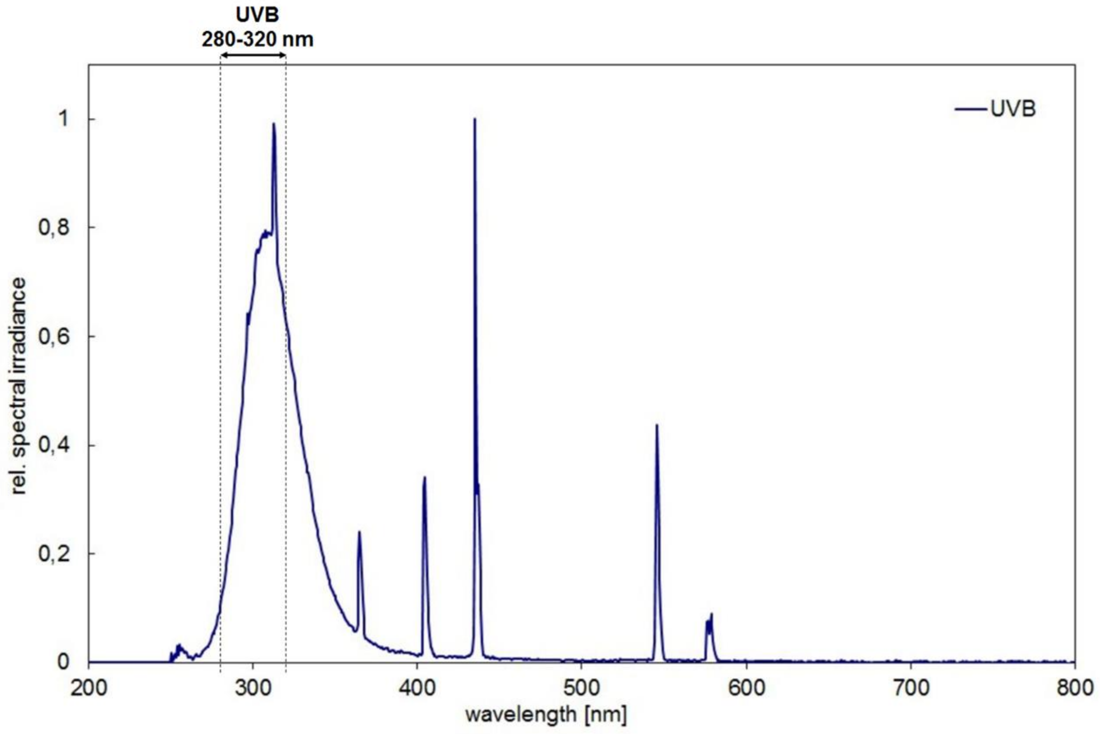

Supplementary Figure S7. UVB spectrum of irradiation chamber BS02. Source: Opsytec Dr. Groebel.

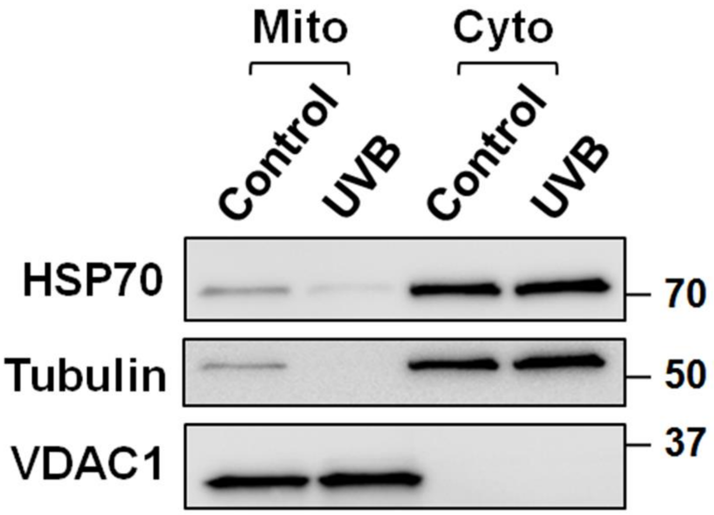

Supplementary Figure S8. Control of cell fractionation by Western blotting. VDAC1, and alpha-tubulin or HSP70, served as markers for the purity of the heavy membrane and cytosolic fractions, respectively, and as markers for equal protein loading.

References

1 Otsu, N. A Threshold Selection Method from Gray-Level Histograms. *IEEE Transactions on Systems, Man, and Cybernetics* **9**, 62-66, doi:10.1109/tsmc.1979.4310076 (1979).
